# Adaptive and Pathological Changes of the Cardiac Muscle in a Mouse Model of Renocardiac Syndrome: The Role of Nestin-Positive Cells

**DOI:** 10.3390/ijms26168100

**Published:** 2025-08-21

**Authors:** Polina A. Abramicheva, Ilya A. Sokolov, Arina A. Druzhinina, Daria M. Potashnikova, Nadezda V. Andrianova, Dmitry S. Semenovich, Vasily N. Manskikh, Ljubava D. Zorova, Elmira I. Yakupova, Ivan M. Vikhlyantsev, Olga S. Tarasova, Dmitry B. Zorov, Egor Y. Plotnikov

**Affiliations:** 1 A.N. Belozersky Institute of Physico-Chemical Biology, M.V. Lomonosov Moscow State University, 119992 Moscow, Russia; ilyasokolov31@gmail.com (I.A.S.); andrianova@belozersky.msu.ru (N.V.A.); 7emenovich@gmail.com (D.S.S.); manskikh@mail.ru (V.N.M.); ljuzor@gmail.com (L.D.Z.); zorov@belozersky.msu.ru (D.B.Z.); 2 School of Biology, M.V. Lomonosov Moscow State University, 119991 Moscow, Russia; druzhininaarinaal@gmail.com (A.A.D.); dpotashnikova@gmail.com (D.M.P.); tarasovaos@my.msu.ru (O.S.T.); 3 V.I. Kulakov National Medical Research Center of Obstetrics, Gynecology, and Perinatology, 117997 Moscow, Russia; 4 Institute of Biomedical Problems of the Russian Academy of Sciences, 123007 Moscow, Russia; elmira.yaku@gmail.com; 5 Institute of Theoretical and Experimental Biophysics, Russian Academy of Sciences, 142290 Pushchino, Russia; ivanvikhlyantsev@gmail.com; 6 Pushchino Branch of the Federal State Budgetary Educational Institution of Higher Education “Russian Biotechnological University (BIOTECH University)”, 142290 Pushchino, Russia; 7 Department of Physiology and Pathology, Faculty of Basic Medicine, M.V. Lomonosov Moscow State University, 119991 Moscow, Russia

**Keywords:** heart, renocardiac syndrome, obstructive nephropathy, nestin-positive cells, cardiac progenitor cells, regeneration

## Abstract

Renocardiac syndrome type 4 (RCS4) is a common comorbid pathology, but the mechanisms of kidney dysfunction-induced cardiac remodeling and the involvement of cardiac progenitor cells (CPCs) in this process remain unclear. The aim of this study was to investigate the structural and functional changes in the cardiac muscle in RCS4 induced by unilateral ureteral obstruction (UUO) and the role of nestin^+^ CPCs in these. Heart function and localization of nestin^+^ cells in the myocardium were assessed using nestin-GFP transgenic mice subjected to UUO for 14 and 28 days. UUO resulted in cardiac hypertrophy, accompanied by an elongation of the QRS wave on the ECG, decreased expression of *Cxcl1*, *Cxcl9*, and *Il1b*, reduced the number of CD11b^+^ cells, and increased in titin isoform parameters, such as T1/MHC and TT/MHC ratios, without changes in fibrosis markers. The number of nestin^+^ cells increased in the myocardium with increased duration of UUO and displayed an SCA-1^+^TBX5^+^ phenotype, consistent with CPCs. Thus, cardiac pathology in RCS4 was manifested by cardiomyocyte hypertrophy with changes in the electrophysiological phenotype of the heart, not accompanied by fibrosis or inflammation. Nestin^+^ cardiac cells retained the CPC phenotype during UUO, and their number increased, which suggests their participation in regenerative processes in the heart.

## 1. Introduction

Renocardiac syndrome type 4 (RCS4) is a pathophysiological condition in which chronic kidney disease (CKD) leads to heart failure [1,2]. Studying the pathogenesis of this disease is of clinical interest, since there are currently difficulties in stratifying the risks of cardiovascular diseases in CKD. In addition, this study is necessary for the development of rational simultaneous therapy for both the heart and kidneys, which requires understanding the mutual influence of these two pathologies. The causes of RCS4 are chronic kidney dysfunction associated with aging, type 2 diabetes mellitus, arterial hypertension, dyslipidemia, and other factors [3]. More than 50% of all patients with CKD have a 10-20 times higher risk of cardiovascular diseases than the same age group without CKD [4]. Unilateral ureteral obstruction (UUO), a classic model of renal tubulointerstitial fibrosis, is a promising model of CKD for cardiology studies. Moreover, this model can be more easily translated to human diseases, as obstructive nephropathy is a common clinical condition that can be irreversible [5].

In response to the development of cardiac dysfunction, a pool of cardiac progenitor cells (CPCs), first identified by Anversa et al. [6], can be activated. This discovery has spurred research into the potential experimental and clinical applications of these cells [7]. In addition to regulating heart embryogenesis, CPCs play a key role in post-injury recovery and can differentiate into various cell types, such as cardiomyocytes, endothelial cells, and smooth muscle cells [8]. Although the existence of progenitor cells in the heart is now well-established, their identification remains challenging because their phenotype can vary depending on the location and developmental stage of the organism.

Key markers of CPCs include KIT proto-oncogene receptor tyrosine kinase (C-KIT), stem cell antigen-1 (SCA-1), platelet-derived growth factor receptor alpha (PDGFRα) [9], and nestin [10]. Initially discovered in neural progenitor/stem cells [11], nestin was later detected in the heart [12], kidney [13], vascular smooth muscle [10], and endothelial cells [14]. During postnatal development, the number of nestin-positive cells in the heart significantly decreases [12]. However, some researchers have reported the presence of neural-derived CPCs even in the adult heart [15,16]. De novo nestin synthesis in cardiac cells plays an important role in reparative fibrosis and angiogenesis, promoting the healing of myocardial tissue damaged by ischemia [17]. However, the involvement of nestin-positive cells in the development of various cardiomyopathies caused by renal dysfunction has not been studied previously. In the present study, we attempted to describe the features of cardiac remodeling in RCS4 induced by UUO of different durations (2 and 4 weeks) and to correlate these changes with the features of nestin-positive cells in the myocardium.

## 2. Results

### 2.1. Structural and Morphological Cardiac Changes in UUO

**Figure 1:**
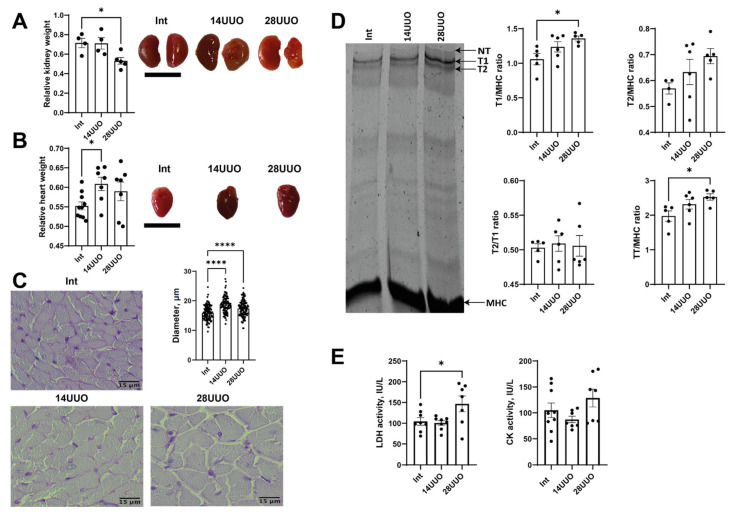
Markers of altered cardiac morphology in RCS 4 of varying duration. Changes in the weight and morphology of the (**A**) left kidney and (**B**) heart in response to obstructive nephropathy in mice. Scale bar: 1 cm. Number of mice per group: (**A**) Int—4; 14UUO—4; 28UUO—5; (**B**) Int—11; 14UUO—8; 28UUO—8. (**C**) Cardiomyocyte diameter assessment in PAS-stained cardiac muscle sections. Scale bar: 15 µm. Number of mice per group: Int—5; 14UUO—5; 28UUO—5. (**D**) Quantitative analysis of sarcomeric titin content in myocardium under RCS4 induced by obstructive nephropathy. The right panel shows a representative electrophoretogram fragment with arrows indicating titin isoforms and MHC bands. Number of mice per group: Int—5; 14UUO—6; 28UUO—6. (**E**) Plasma LDH and CK enzyme activity in obstructive nephropathy of different durations. Number of mice per group: Int—13; 14UUO—12; 28UUO—12. * *p* < 0.05, **** *p* < 0.0001 statistically significant differences compared to the control (“Int”) group (one-way ANOVA, Dunnett’s post hoc test).

**Figure A1:**
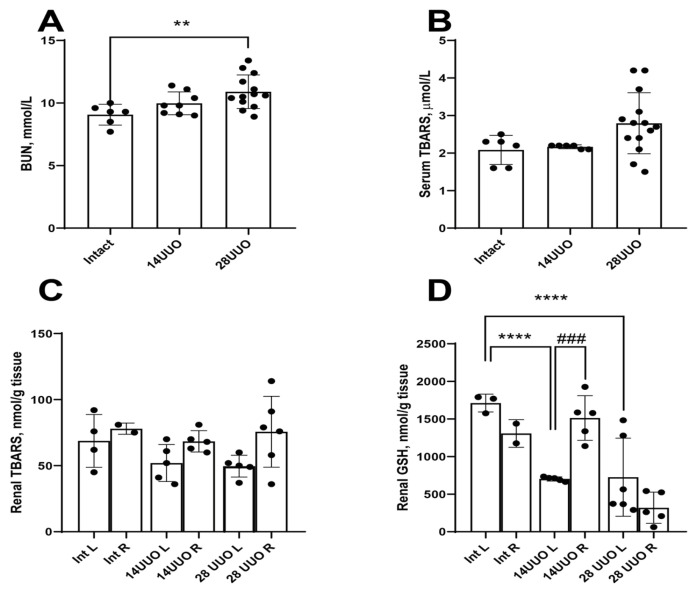
Renal damage and oxidative stress markers from mice with different UUO durations. BUN (**A**), serum (**B**), renal (**C**) TBARS, and renal glutathione levels (**D**) from mice subjected to UUO of different durations. ** *p* < 0.01, **** *p* < 0.0001—statistically significant differences compared to the “Int” group (one-way ANOVA, Dunnett’s post hoc test), ### *p* < 0.001—statistically significant differences compared to the “14UUO L” group (unpaired *t*-test). L—left (obstructive) kidney, R—right (intact) kidney, BUN—blood urea nitrogen, TBARS—thiobarbituric acid reactive substances, GSH—glutathione.

The mass of the obstructed kidney, accepted as a morphological parameter of progressive nephrosclerosis, showed progressive reduction correlating with UUO duration (Figure 1A), as compared to the intact contralateral kidney. This decrease in mass implies progressive renal dysfunction [18]. Additional markers of renal damage and oxidative stress are shown in Figure A1. Simultaneously, obstructive nephropathy is associated with a slight increase in heart mass (Figure 1B). These observations suggest the development of myocardial hypertrophy, supported by a histological examination of cardiac tissue sections stained with periodic acid–Schiff (PAS) reagent (Figure 1C).

We explored the effect of RCS4 on the cardiac levels of titin, which plays an important role in the contraction of striated muscle. A significant increase was observed in the ratio of intact titin-1 (T1, represented by N2B, N2BA, the NT isoform found in mammalian striated muscles [19]) to myosin heavy chains (MHC), and the ratio of total titin (TT, comprising T1 + proteolytic T2 fragment) to MHC in the 28UUO group compared to Int controls (Figure 1D). A trend toward an increased T2/MHC ratio was noted, while the T2/T1 ratio remained unchanged across all obstruction time points (Figure 1D).

Finally, we assessed the activity of the plasma enzymes associated with cardiac dysfunction. In response to 28-day ureteral obstruction, lactate dehydrogenase (LDH) activity increased, whereas creatine kinase (CK) activity remained unchanged in both UUO groups (Figure 1E).

### 2.2. Hemodynamic and ECG Indices in UUO

**Figure 2:**
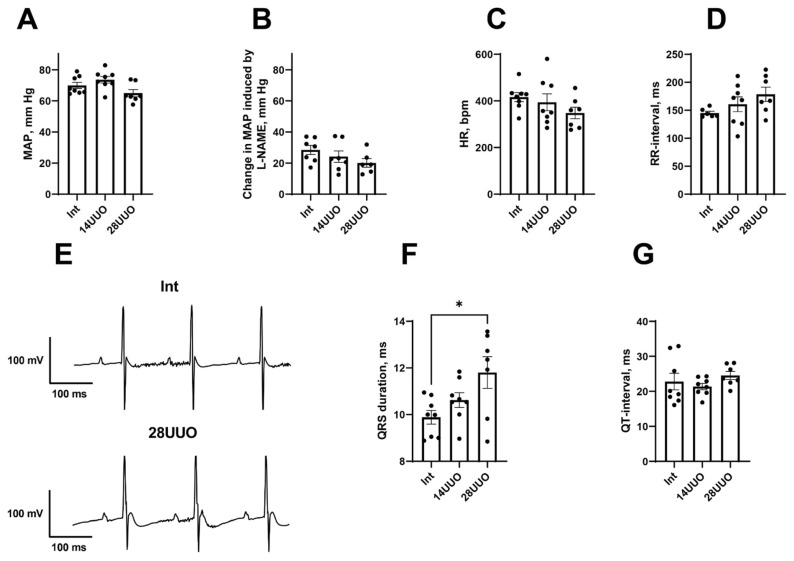
Hemodynamic variables and ECG parameters in UUO. (**A**–**D**): MAP (**A**), MAP response to L-NAME administration (**B**), HR (**C**), and RR-interval duration (**D**) in the three groups of mice. (**E**): Fragments of representative ECG recordings showing QRS prolongation in 28UUO group compared to the Int group. (**F**,**G**): durations of QRS complex and QT-interval in the three groups of mice. Number of mice per group: Int—8; 14UUO—8; 28UUO—7. * *p* < 0.05—statistically significant difference compared to the Int group (one-way ANOVA, Dunnett’s post hoc test).

Mean arterial pressure (MAP) remained unchanged at 14 and 28 days of UUO (Figure 2A). In addition, the MAP response to NO synthase inhibitor N-nitro-L-arginine methyl ester (L-NAME) did not differ between the experimental groups, proposing the absence of systemic endothelial dysfunction in UUO (Figure 2B). We also did not observe any changes in heart rate (HR, Figure 2C) or, accordingly, in RR interval duration (Figure 2D) in the UUO of either duration. However, the duration of the QRS complex was increased in a 28-day-long UUO group compared to the Int group (Figure 2E,F). Along with that, the duration of the QT interval remained unchanged in both experimental groups (Figure 2G).

### 2.3. In RCS4 Induced by UUO, Cardiac Muscle Fibrosis Does Not Occur

**Figure 3:**
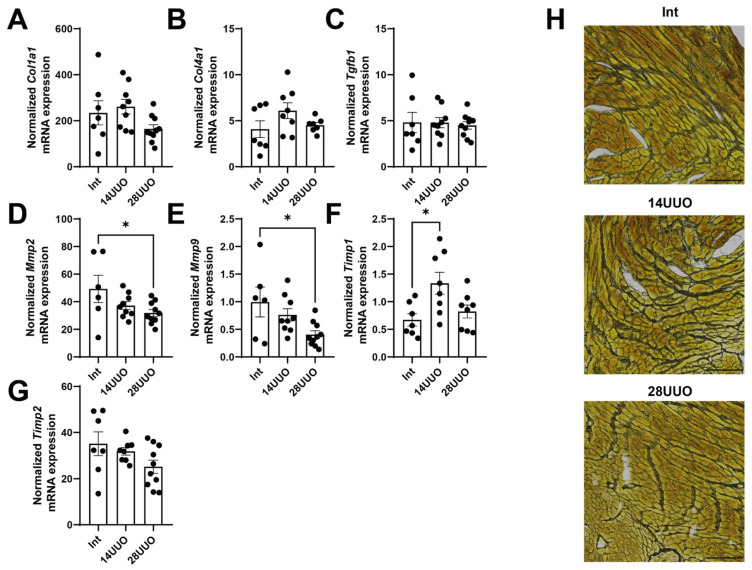
Expression of fibrosis markers (**A**–**C**) and remodeling markers (**D**–**G**) in the cardiac muscle in response to RCS4. Number of mice per group: (**A**–**G**) Int—7; 14UUO—9; 28UUO—10. (**H**) Staining of cardiac muscle sections for reticulin using Gordon–Sweet method. Scale bar: 100 µm. Number of mice per group: Int—5; 14UUO—5; 28UUO—5. * *p* < 0.05—statistically significant differences compared to the “Int” group (one-way ANOVA, Dunnett’s post hoc test). Exact *p*-values: (**D**): Int vs. 28UUO—*p* = 0.0381; (**E**): Int vs. 28UUO—*p* = 0.0168; (**F**): Int vs. 14UUO—*p* = 0.0115.

We evaluated the expression of key fibrosis marker genes in the heart and demonstrated that, under RCS4 conditions, the expression of extracellular matrix (ECM) components—type I and IV collagens (*Col1a1* and *Col4a1*, respectively)—as well as *Tgfb1*, remained unchanged (Figure 3A–C, respectively). However, we found that the expression of regulators of epithelial–mesenchymal transition and mediators of cardiac fibrosis, matrix metalloproteinases *Mmp2* (Figure 3D), and *Mmp9* (Figure 3E) decreased with prolonged UUO. This is consistent with the increased expression of the tissue inhibitor of metalloproteinases (*Timp1*), which suppresses the activity of these metalloproteinases (Figure 3F), while the expression of *Timp2* did not change (Figure 3G). The absence of profibrotic changes was further confirmed by histochemical analysis of cardiac muscle sections stained for reticulin (Figure 3H). Thus, the lack of significant fibrosis and cardiac tissue remodeling suggests the presence of an alternative mechanism driving cardiac dysfunction, leading to myocardial hypertrophy.

### 2.4. Expression of Proinflammatory Cytokines and Macrophage Content in the Heart Following UUO of Different Durations

**Figure 4:**
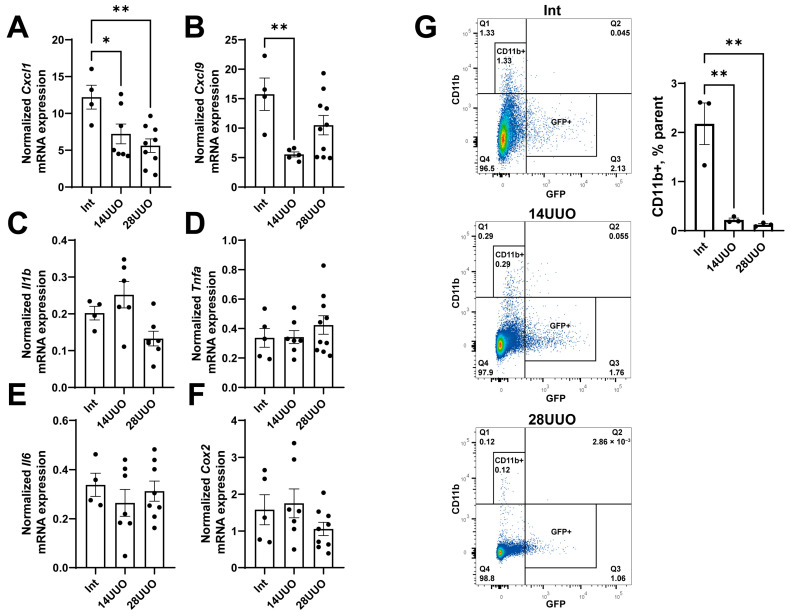
Expression profile of proinflammatory chemokines (**A**,**B**), cytokines (**C**–**E**), and prostaglandin synthesis regulator (**F**) in the heart at different time points of UUO. Number of mice per group: (**A**–**F**) Int—5; 14UUO—7; 28UUO—10. (**G**) Analysis of CD11b^+^ macrophage content in the heart at different stages of UUO. Representative dot plots show the percentage of cells in the parent gate. Number of mice per group: Int—3; 14UUO—3; 28UUO—3. * *p* < 0.05, ** *p* < 0.01—statistically significant differences compared to the “Int” group (one-way ANOVA, Dunnett’s post hoc test). Exact *p*-values: (**A**): Int vs. 14UUO—*p* = 0.0364, Int vs. 28UUO—*p* = 0.005; (**B**): Int vs. 14UUO—*p* = 0.0078; (**G**): Int vs. 14UUO—*p* = 0.0024, Int vs. 28UUO—*p* = 0.0019.

While increasing the UUO duration, the expression of the proinflammatory chemokines *Cxcl1* and *Cxcl9* decreased in the heart (Figure 4A,B, respectively). The mRNA levels of proinflammatory cytokines (*Il1b*, *Tnfa*, *Il6*) and the key regulator of prostaglandin synthesis, cyclooxygenase-2 (*Cox2*), remained unchanged (Figure 4C–F, respectively). These findings were consistent with the flow cytometry data showing that the number of CD11b^+^ macrophages significantly decreased in the heart as early as day 14 of UUO (Figure 4G). Furthermore, flow cytometry analysis (Figure 4G) indicated that the CD11b^+^ cells did not belong to nestin^+^ GFP-expressing cells.

### 2.5. Analysis of Nestin-Positive Cell Count in the Hearts of Mice with RCS4 Induced by Obstructive Nephropathy

**Figure 5:**
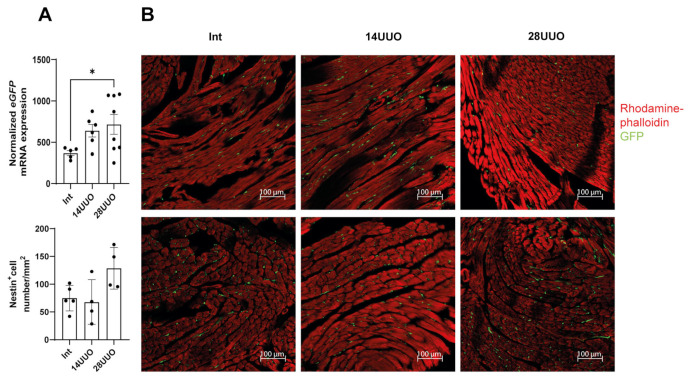
Effect of UUO on GFP-positive cells in the heart. (**A**) *eGFP* mRNA levels analysis. Number of mice per group: Int—7; 14UUO—7; 28UUO—8. (**B**) Distribution and number of nestin-positive cells in cryosections of mouse hearts with different UUO durations, stained with rhodamine–phalloidin. Upper panel (**B**): longitudinal sections of the left ventricle, lower panel (**B**): transverse sections. Rhodamine–phalloidin stains the actin cytoskeleton, and GFP labels nestin-positive cells. Scale bar: 100 μm. Number of mice per group: Int—5; 14UUO—4; 28UUO—4. * *p* < 0.05—statistically significant differences compared to the “Int” group (one-way ANOVA, Dunnett’s post hoc test). For each sample, ten fields of view were obtained.

We found that prolonged obstruction led to increased *eGFP* mRNA expression levels in the heart (Figure 5A), indicating an increase in the number of GFP-expressing cells or elevated nestin expression. Confocal microscopy revealed that nestin-positive GFP-expressing cells were localized between cardiomyocytes in both longitudinal and transverse cryosections of mouse hearts from different experimental groups, stained with rhodamine–phalloidin. Notably, the distribution pattern and spatial localization of these cells remained unchanged, even with prolonged UUO duration (Figure 5B).

### 2.6. Analysis of Nestin Colocalization with Markers of Different Cardiac Cell Types

**Figure 6:**
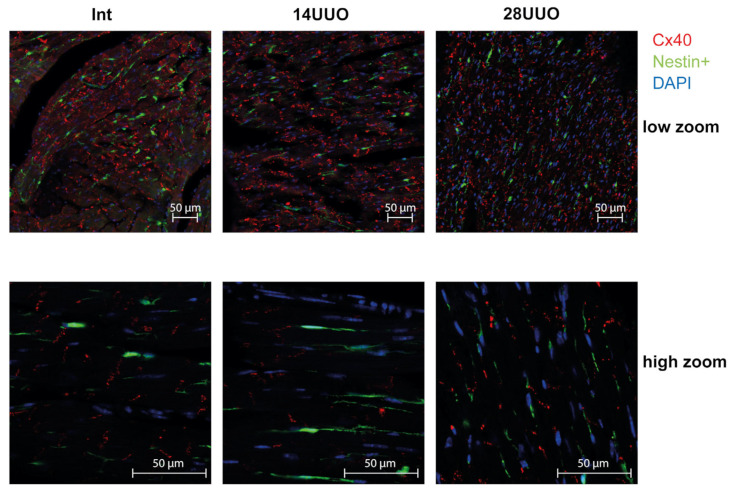
Representative confocal images of left ventricle cryosections from mice with different UUO durations, stained with DAPI and antibodies against connexin 40. Scale bar: 50 μm. Number of mice per group: Int—5; 14UUO—4; 28UUO—4. For each sample, ten fields of view were obtained.

**Figure 7:**
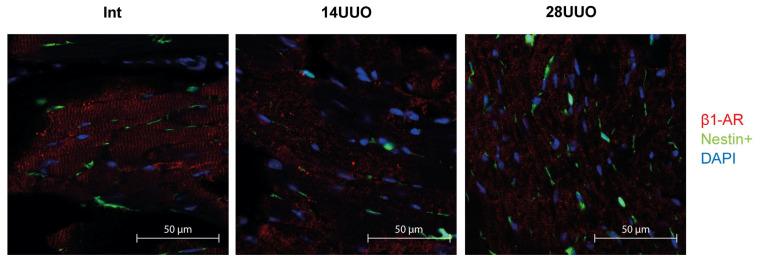
Representative confocal images of left ventricle cryosections from mice with different UUO durations, stained with DAPI and antibodies against β1-adrenergic receptor. Scale bar: 50 μm. Number of mice per group: Int—5; 14UUO—4; 28UUO—4. For each sample, ten fields of view were obtained.

**Figure 8:**
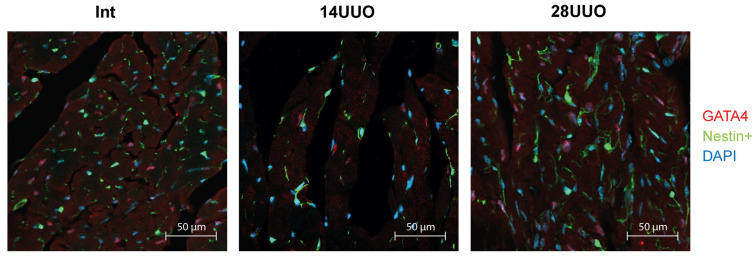
Representative confocal images of left ventricle cryosections from mice with different UUO durations, stained with DAPI and antibodies against GATA4. Scale bar: 50 μm. Number of mice per group: Int—5; 14UUO—4; 28UUO—4. For each sample, ten fields of view were obtained.

**Figure 9:**
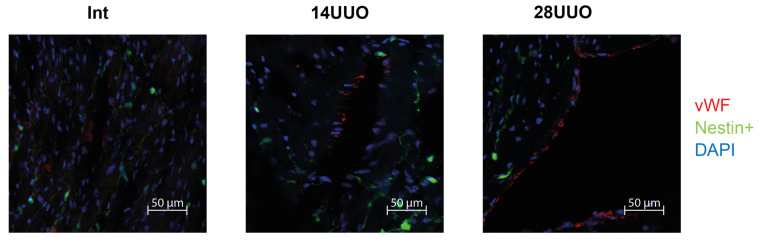
Representative confocal images of left ventricle cryosections from mice with different UUO durations, stained with DAPI and antibodies against von Willebrand factor. Scale bar: 50 μm. Number of mice per group: Int—5; 14UUO—4; 28UUO—4. For each sample, ten fields of view were obtained.

We analyzed the colocalization of markers from various cardiac cell types with nestin-positive cells. Our results demonstrated that GFP did not colocalize with the cardiomyocyte markers connexin 40 (Figure 6) and β1-adrenergic receptor (β1-AR) (Figure 7). Similarly, no colocalization was observed with either the cardiogenesis and CPC marker GATA4 (Figure 8) or the endothelial cell marker von Willebrand factor (vWF) (Figure 9). These findings indicate that nestin-positive cells are neither cardiomyocytes nor endothelial cells. Regarding CPCs, this cell population shows heterogeneity in its marker expression profile [20]. A subset of CPCs is known to express the transcription factor GATA4 in the adult myocardium. Our results demonstrate that nestin-positive cells lack GATA4 expression and likely represent a distinct CPC population.

### 2.7. GFP^+^ Cardiac Cells Express Cardiac Progenitor Cell Markers

**Figure 10:**
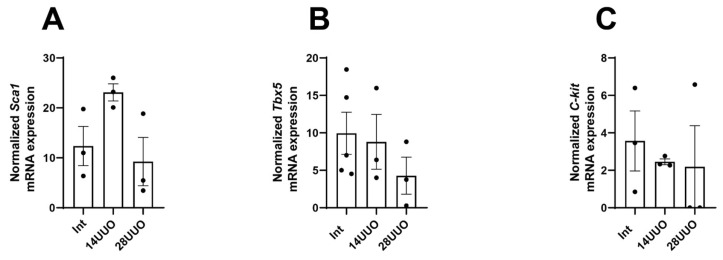
mRNA expression of CPC markers *Sca-1* (**A**), *Tbx5* (**B**), and *C-kit* (**C**) in GFP^+^ cardiac cells at different UUO time points. Number of mice per group: (**A**) Int—3; 14UUO—3; 28UUO—3; (**B**) Int—5; 14UUO—3; 28UUO—3; (**C**) Int—3; 14UUO—3; 28UUO—3. One-way ANOVA, Dunnett’s post hoc test.

To isolate a pure population of nestin-positive cells from the myocardium, we performed fluorescence-activated cell sorting (FACS) of GFP-positive cells from heart homogenates obtained from mice with obstructive nephropathy. The sorted cells were then analyzed for the expression of CPC marker genes. The GFP-negative cell population was sorted and examined in parallel. We found that GFP^+^ cardiac cells exhibited a significantly higher expression of CPC markers (*Sca-1*, *Tbx5*, and *C-kit*) compared to GFP^−^ cells. Notably, their expression levels remained stable across different UUO time points (Figure 10A–C).

## 3. Discussion

Patients with CKD exhibit a high prevalence of cardiovascular complications, particularly arrhythmias and ultimately, sudden cardiac death. Therefore, elucidating the structural and functional remodeling of the heart, including the potential for CPC-mediated reparative fibrosis and angiogenesis, is critical for understanding the pathogenesis of RCS4. In experimental in vivo settings, comparable pathological changes can be reproduced by renal artery occlusion [21] and other conventional CKD models [22,23]. In this study, we demonstrated the development of cardiac hypertrophy in GFP–nestin mice with progressive UUO duration (Figure 1B,C), which occurred without changes in blood pressure and cardiac chronotropism. This data has been supported by previously published studies on this murine UUO model [23,24]. In general, such pathological changes recapitulate the clinical phenotype observed in uremic patients, characterized by left ventricular hypertrophy, ventricular dilation, and systolic dysfunction [25].

Apparently, changes in titin content may provide critical insights into the morphofunctional state of the myocardium in obstructive nephropathy. Titin, as the third elastic sarcomeric filament, was found to be critical for proper cardiac function [26]. We measured the T1/MHC, T2/MHC, T2/T1, and TT/MHC ratios, which can reflect titin synthesis and turnover in muscle cells [19]. We observed an increase in the T1/MHC and TT/MHC ratios (Figure 1D) in the 28UUO group. This data may indirectly support evidence of myocardial hypertrophy during UUO. It is worth noting that the TT/MHC ratio is usually a stable parameter in the heart, even under pathological conditions (e.g., aortocaval fistula surgery [27], heart failure with preserved ejection fraction [28], and dilated cardiomyopathy [29]). Our data is the first observation of an increase in this ratio during RCS4, which may indicate hypertrophic disorders at the level of sarcomere proteins.

We found that morphological changes in the cardiac muscle in UUO were accompanied by the prolongation of the QRS complex (Figure 2F). In humans, such an ECG alteration is often attributed to the increase in ventricular activation time due to the left bundle branch block [30]. However, in mice, a QRS prolongation may be observed along with normal ventricular conduction velocity but increased myocardial mass [31]. Presumably, the QRS prolongation in our 28UUO mice resulted from cardiac hypertrophy.

Finally, we observed a significant increase in plasma LDH activity in 28UUO (Figure 1E), suggesting a possible cytolysis of renal and cardiac tissues due to the development of RCS4. However, total LDH activity in plasma alone cannot differentiate renal or cardiac tissue damage. Therefore, for a comprehensive understanding of this pathology, these laboratory parameters had to be interpreted in conjunction with a histopathological examination of both cardiac and kidney tissues in mice.

Notably, cardiac hypertrophy in mice with UUO occurred without ECM deposition (Figure 3A–C,H). The absence of myocardial fibrosis may be attributed to the UUO duration being insufficient to initiate this process. Alternatively, CPCs might suppress fibrosis progression through paracrine secretion of various factors (e.g., HGF and IGF-1 [32]), thereby inhibiting TGFβ signaling, ECM deposition, and histone deacetylase activation [33,34]. We observed decreased mRNA expressions of *Mmp2* and *Mmp9* (Figure 3D and Figure 3E, respectively) alongside increased *Timp1* expression (Figure 3F) in cardiac tissue in the 28UUO group. The MMP/TIMP balance is controlled by an intricate regulatory network, where alterations in their activity and expression exhibit context-dependent effects. It is well-established that MMP9 activity increases significantly during the acute post-infarction period [35], while *Timp1* expression is elevated in various cardiac pathologies [36,37]. Although MMP9 inhibition (by aldosterone antagonists and ACE inhibitors [38]) represents a therapeutic approach for heart failure, atherosclerosis, hypertension, and myocardial infarction, patients with congestive heart failure demonstrate reduced *Mmp9* mRNA levels in cardiac biopsies [39]. Conversely, reduced *Mmp2* expression may lead to disrupted collagen turnover, impaired cardiac metabolism, and ultimately fibrosis progression as observed in diabetic cardiomyopathy [40]. Furthermore, MMP2-knockout mice exhibit impaired cardiac remodeling and mitochondrial dysfunction [41]. Thus, both increased and decreased expression of MMPs and TIMPs can contribute to cardiac tissue remodeling and acute heart failure development. However, in our study, the observed downregulation of *Mmp2* and *Mmp9* may confer cardioprotective effects. There is evidence that *Mmp9* deletion reduces post-infarction macrophage infiltration, thereby preventing collagen accumulation, while promoting neovascularization and remodeling of the left ventricle [42].

These findings are consistent with the observed reduction in CD11b^+^ cells (primarily macrophages) in both the 14UUO and 28UUO groups (Figure 4G). As a key mediator of innate immunity, decreased CD11b^+^ cell infiltration may reflect diminished chemokine secretion due to UUO (Figure 4A,B), consequently attenuating inflammatory responses. Specifically, CXCL1 and CXCL9 exert proinflammatory effects through distinct CXCR receptor isoforms, activating neutrophils and stimulating T-lymphocytes [43]. Studies demonstrate that inhibition of either CXCL1 or its receptor CXCR2 can attenuate cardiac inflammation, hypertrophy, and fibrosis [44]. While reduced *Cxcl9* expression similarly indicates suppressed myocardial inflammatory response, patients with acute coronary syndrome show decreased peripheral blood levels of this chemokine—likely due to monocyte recruitment to infarct zones, where CXCL9 serves as a potent chemoattractant [45].

We observed an increased *eGFP* expression that correlated with UUO duration (Figure 5A). This upregulation may reflect either the expansion of nestin-expressing cell populations (in these transgenic mice with GFP under the nestin promoter) or enhanced nestin gene activation. Elevated cardiac nestin expression is a recognized feature of various cardiomyopathies and may be involved in myocardial remodeling. Though predominantly expressed in embryonic stem cells during development, nestin can be re-expressed in mature cells following injury, serving as a dual marker for both regenerative processes and pathological fibrosis [46,47]. In a murine model of Duchenne muscular dystrophy, the transplantation of nestin-positive cells into myocardial tissue stimulated the proliferation of endogenous CPCs expressing this marker, thereby preventing the development of dilated cardiomyopathy [48]. In adult rodent hearts, nestin-positive cells were detected occupying the areas between cardiomyocytes (Figure 5B). Following myocardial infarction, these cells migrate to the scar region during reparative fibrosis [10,16,49]. Several studies report nestin expression in various cardiac cell types: CPCs (constitutive expression) [50], mature cardiomyocytes (de novo synthesis after myocardial infarction) [51], mature ventricular fibroblasts [12], endothelial cells (de novo synthesis during myocardial infarction/hypertension [14,52] and other cell types.

Our studies in intact mice and UUO-induced RCS4 models demonstrate that nestin-positive cells lack expression of the following markers: the cardiomyocyte markers Cx40 and β1-AR (Figure 6 and Figure 7, respectively), the cardiogenic marker GATA4 (Figure 8), and the vascular endothelial marker vWF (Figure 9). However, they exhibit a TBX5^+^SCA-1^+^ phenotype (Figure 10A,B). While SCA-1 was originally identified in hematopoietic stem cells, it also marks CPCs in the heart. The SCA-1^+^ cardiac cell population displays a mesenchymal phenotype (PDGFRα^+^CD44^+^CD90^+^) and may differentiate into cardiomyocytes and endothelial and smooth muscle cells [53]. TBX5, a transcription factor regulating cardiogenesis, also controls nestin gene expression [54,55] and persists in the adult CPC subpopulation [8].

**Figure 11:**
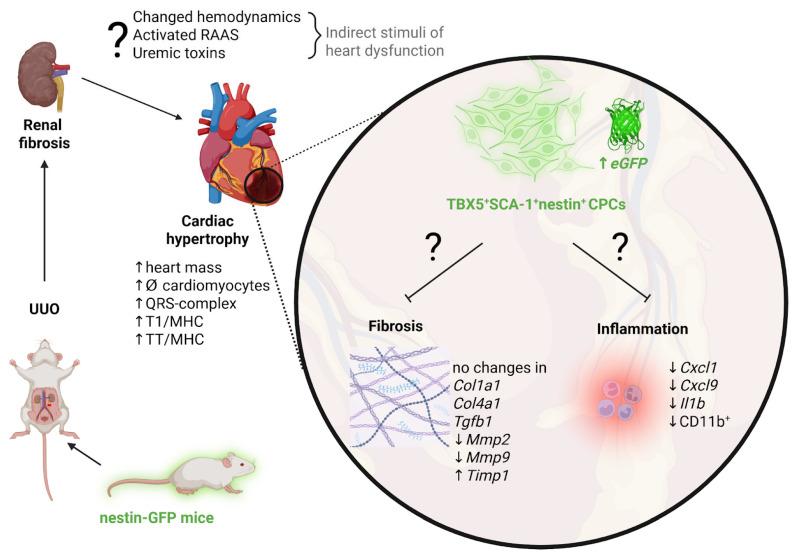
Proposed mechanisms driving cardiac hypertrophy progression and the potential involvement of nestin-positive cells in adaptive cardiac remodeling in UUO-mediated RCS4. Created in https://BioRender.com.

Thus, we propose that nestin^+^TBX5^+^SCA-1^+^ cells represent a CPC population that may exert regenerative potential during UUO-induced RCS4. However, the exact mechanism underlying this compensatory response that prevents cardiac fibrosis in RCS4 is challenging to elucidate. We hypothesize that UUO-mediated cardiac hypertrophy may be caused by the activation of the renin–angiotensin–aldosterone system, hemodynamic alterations, and accumulation of uremic toxins in acute/chronic kidney disease [56]. The development of cardiac hypertrophy may stimulate the activation of the CPC pool, which exerts its regenerative potential by suppressing fibrosis and the inflammatory response. The signaling pathways mediating these protective mechanisms in the myocardium under UUO-induced RCS4 remain to be elucidated in future studies. A potential mechanism for the progression of cardiac dysfunction in primary CKD and the role of nestin-positive cells in this process is illustrated in Figure 11.

To further elucidate the pathogenesis of RCS4 in the UUO model and the role of nestin-positive cells in this process, future studies should address the limitations of our work, including an assessment of long-term outcomes following sustained ureteral obstruction and the use of functional CPC differentiation assays. In this study, experiments were performed on male subjects because CKD is more prevalent in men [57,58]. Nevertheless, further research into sex differences in RCS4 progression remains warranted.

## 4. Materials and Methods

### 4.1. Animals

Experiments were performed on 2-month-old male nestin–GFP transgenic mice [59]. The nestin–GFP mice were generated [60] and kindly provided by Grigori Enikolopov. The study was performed according to the guidelines of the Declaration of Helsinki and approved by the A.N. Belozersky Institute of Physico-Chemical Biology Lomonosov Moscow State University (protocol No. 009-4/03/2024). All procedures were executed in accordance with the “Animal Research: Reporting of In Vivo Experiments” (ARRIVE) guidelines.

### 4.2. Experimental Design

Animals were divided into 3 groups: intact (Int), with 14-(14UUO), and 28-day unilateral ureteral obstruction (28UUO). UUO was performed as previously described [5]. Sample size and additional descriptive statistics of each experimental group for all assays are included in Table A1 and Table A2, respectively. Briefly, mice were anesthetized with isoflurane and placed supine on a heating pad, and the left ureter was visualized, double ligated with 4-0 silk, and dissected. Mice were sacrificed after 14 or 28 days of ureteral obstruction.

### 4.3. Morphometric Analysis

The hearts of mice from each experimental group were isolated, weighed, and photographed. Additionally, the kidneys were photographed to confirm the presence of marked obstructive nephropathy. Cardiac hypertrophy was assessed using the following formula:

(heart weight/body weight) × 100%

### 4.4. ECG and Blood Pressure Recording

Mice were anesthetized with chloral hydrate (480 mg/kg, i.p.) and placed in a supine position on a heating plate to maintain body temperature at 36–37 °C. For ECG recording, two needle electrodes (26 G) were placed subcutaneously above the right clavicle and in the left hypochondrium (along the standard lead II); the reference electrode was inserted under the right thigh skin. After a 15–20 min ECG recording, the electrodes were removed, and a polyethylene catheter (PE5-PE50) connected to a BLPR2 pressure transducer (World Precision Instruments, Sarasota, FL, USA) was inserted into the left common carotid artery for arterial blood pressure measurements. To avoid blood clotting, the catheter was continuously flushed with heparinized saline (50 U/mL) using a Syringe pump (model 341, SAGE Instruments, Watsonville, CA, USA), with the infusion rate at 0.09 mL/h. After a 15 min stabilization period, a non-selective inhibitor of nitric oxide synthases, L-NAME (Chem-Impex International, Wood Dale, IL, USA), was administered (40 mg/kg, i.p.), and the second 25 min-long recording was obtained. Finally, the anesthetized mouse was euthanized by decapitation, and a trunk blood sample was obtained.

The signals were amplified and continuously sampled at 1000 Hz using a USB-6211 analog-to-digital converter (USB-6211, National Instruments, Austin, TX, USA). Data acquisition and processing were performed using original software written in LabView 2011 (National Instruments, USA) [61]. The last 5 min interval of ECG recording was exported to a binary file and then imported into LabChart 7.3.4 with ECG Analysis Module 2.3.2 (ADInstruments, Dunedin, New Zealand). ECG parameters were estimated for every cardiac cycle and then averaged for all cycles. The blood pressure signal was processed beat-to-beat to estimate values of MAP and HR. Then MAP and HR were averaged in 3 min-long intervals: before and 20 min after L-NAME administration.

### 4.5. Histochemical Analysis

Paraformaldehyde-fixed hearts (*n* = 5 for each experimental group) were embedded in paraffin and cut into 4 μm sections. For PAS staining, sections were oxidized with 0.5% periodic acid for 5 min, rinsed in distilled water, stained with Schiff reagent for 15 min, and rinsed in running tap water for 5 min. The nuclei were counterstained with alum hematoxylin. Staining with Schiff’s reagent is an effective method for microscopic analysis of left ventricular hypertrophy. This technique selectively highlights the ECM deposited around each muscle fiber, clearly delineating individual cardiomyocytes. Consequently, it allows for accurate measurement of cardiomyocyte diameter in transverse myocardial sections. Diameters of 100 cardiomyocytes per slice on ten images randomly selected for analysis were measured in a blinded fashion by FIJI software 1.54p (NIH, Bethesda, MD, USA). Gordon–Sweet’s reticulin staining was performed on deparaffinized sections, which were incubated in 1% potassium permanganate solution for 5 min, rinsed in tap water, bleached in 1% oxalic acid solution, rinsed again in tap water, and incubated with 2.5% ferric alum solution for 30 min. After the last treatment, sections were washed in three changes of distilled water, placed in a Coplin jar of ammonium silver solution for 13 s, quickly rinsed in distilled water, and reduced in a 10% aqueous formalin solution for 2 min. After washing in tap water, the slides were treated with 5% sodium thiosulfate solution for 3 min. All stained heart slices were viewed with the CELENA^®^ X High Content Imaging System (Anyang-si, Republic of Korea).

### 4.6. Titin Electrophoresis

SDS-polyacrylamide gel electrophoresis (SDS-PAGE) was performed according to the method described in [62], using vertical 0.5% agarose gels with 2.3% polyacrylamide. To minimize degradation of the high-molecular-weight titin isoforms, homogenized left ventricular samples from mice were incubated for 10 min at 40 °C in a solution containing 10 mM Tris-HCl, 1.5% SDS, 10% glycerol, 2% β-mercaptoethanol, protease inhibitor cocktail complete ultra tablets (Roche Diagnostics, Meylan, France), and 1 mM sodium orthovanadate. SDS-PAGE was carried out using the Helicon VE-10 system (Helicon Company, Moscow, Russia) at a constant current of 3 mA. After separation, the gels were stained with a 1:1 mixture of Coomassie Brilliant Blue G-250 and R-250. Gel imaging was performed using the ChemiDoc MP Imaging System (Bio-Rad Laboratories, Inc., Hercules, CA, USA) in GelStar mode. The target bands (T1 (including N2B, N2BA, and NT-isoforms), T2, and MHC) were quantified using ImageJ 1.54 software (NIH, Bethesda, MD, USA). Band intensities were normalized to MHC within each lane.

### 4.7. Plasma LDH and CK Activity

Blood was collected from mice immediately after decapitation into heparinized tubes (50 U/mL heparin). Samples were centrifuged at 1500× *g* for 10 min at 4 °C to obtain plasma, which was stored at −70 °C until analysis. Plasma CK and LDH activities were measured using modified kinetic spectrophotometric assays [63,64]. Enzyme activities were determined by monitoring the absorbance changes at 340 nm (37 °C) using a Zenith 3100 multimode microplate reader (Anthos, Salzburg, Austria).

### 4.8. Real-Time PCR

The mRNA expression levels of GFP (*eGFP*), fibrosis-related genes (*Col1a1*, *Col4a1*, *Tgfb1*), and extracellular matrix regulators, including matrix metalloproteinases (*Mmp2*, *Mmp9*) and their inhibitors (*Timp1*, *Timp2*), as well as inflammatory response genes (*Cxcl1*, *Cxcl9*, *Il1b*, *Il6*, *Tnfa*, *Cox2*) and cardiac progenitor cell markers (*C-kit*, *Sca-1*, *Tbx5*), were quantified in mice at various time points following UUO. The 60S acidic ribosomal protein (*Rplp0*) served as the housekeeping gene for normalization. Total RNA was isolated from heart tissue and FACS-sorted GFP^+^ and GFP^−^ cells using TRIzol reagent (Thermo Fisher Scientific, Carlsbad, CA, USA). Following homogenization and chloroform centrifugation, the aqueous phase containing nucleic acids was precipitated with 75% ethanol. Further purification of RNA from heart tissue, including DNAse treatment, was performed using the RNeasy Mini Kit (QIAGEN, Hilden, Germany), while the RNA from sorted cells was extracted with the Zymo Quick-RNA Microprep Kit (Zymo Research, Orange, CA, USA). Reverse transcription was performed using the MMLV RT Kit (Evrogen, Moscow, Russia) according to the manufacturer’s protocol. Real-time PCR was performed using the Bio-Rad CFX96 Real-Time PCR System (Bio-Rad Laboratories, Inc., Hercules, CA, USA) and 5X qPCRmix-HS master mix (Evrogen, Russia). Primers (DNA-synthesis, Moscow, Russia) were designed using the cloud-based Benchling platform (San Francisco, CA, USA) and Primer-BLAST (NCBI/NLM, Bethesda, MD, USA). Primer sequences are provided in Table A3.

### 4.9. Confocal Microscopy

The hearts isolated from the mice of different experimental groups were washed with PBS and fixed in formalin for 24 h. After rinsing with water, the samples were processed through a sucrose gradient (10%, 20%, 30%) and embedded in OCT medium (Tissue-Tek O.C.T. Compound, Torrance, CA, USA before being flash-frozen in nitrogen vapor. Cryosections (10 μm thick) were prepared using a cryostat (Leica, Wetzlar, Germany). Myocardial sections were incubated overnight at 4 °C in PBS containing 0.1% Triton X-100 and 1 μM rhodamine–phalloidin (R145, Invitrogen, Carlsbad, CA, USA) to visualize the actin cytoskeleton. Additionally, sections were co-stained with primary antibodies against cx40 (1:100, rabbit, CX40-S, Alpha Diagnostic International, San Antonio, TX, USA), β1-AR (1:100, rabbit, β1-AR PA1-049, Invitrogen, Carlsbad, CA, USA), GATA4 (1:400, rabbit, PA1-102, Invitrogen, Carlsbad, CA, USA), or vWF (1:100, rabbit, ab6994, Abcam, Waltham, MA, USA). Goat anti-rabbit secondary antibodies conjugated to Cy5 (1:100, 111-175-144, Jackson ImmunoResearch, Ely, UK) were used for detection. The prepared sections were mounted in a Fluroshield mounting medium (Sigma, Burlington, MA, USA) or LumiMount^®^ DAPI (Lumiprobe, Moscow, Russia) for nuclear staining. The stained samples were imaged using an LSM 900 inverted confocal microscope (Carl Zeiss, Oberkochen, Germany) using C-Apochromat 10×/0.45 W or C-Apochromat 63×/1.20 W Korr M27 objectives. GFP fluorescence was detected at an excitation wavelength of 488 nm (laser power 4.5%) and an emission at 500–530 nm, with a detector gain of 550 V. Rhodamine–phalloidin was acquired at 543 nm excitation (laser power 5%) and 616–700 nm emission (detector gain 580 V). For connexin 40, β1-adrenergic receptor, GATA4, or vWF acquisition, Cy5 was excited at 543 nm (laser power 8%) and 616–700 nm emission (detector gain 600–800 V, depending on the antigen). DAPI was acquired at 405 nm excitation (laser power 1%) and 410–483 nm emission (detector gain 600 V). For all images, the pinhole was 1 AU for all tracks and wavelengths and a scan speed of 7, averaging 2. The acquired images were analyzed using ZEN 3.1 blue edition software (Carl Zeiss, Oberkochen, Germany). The number of nestin^+^ cells in cardiac tissue sections stained with rhodamine–phalloidin was quantified across all experimental groups using ImageJ 1.54 software (NIH, Bethesda, MD, USA).

### 4.10. Flow Cytometry and Cell Sorting

**Figure A3:**
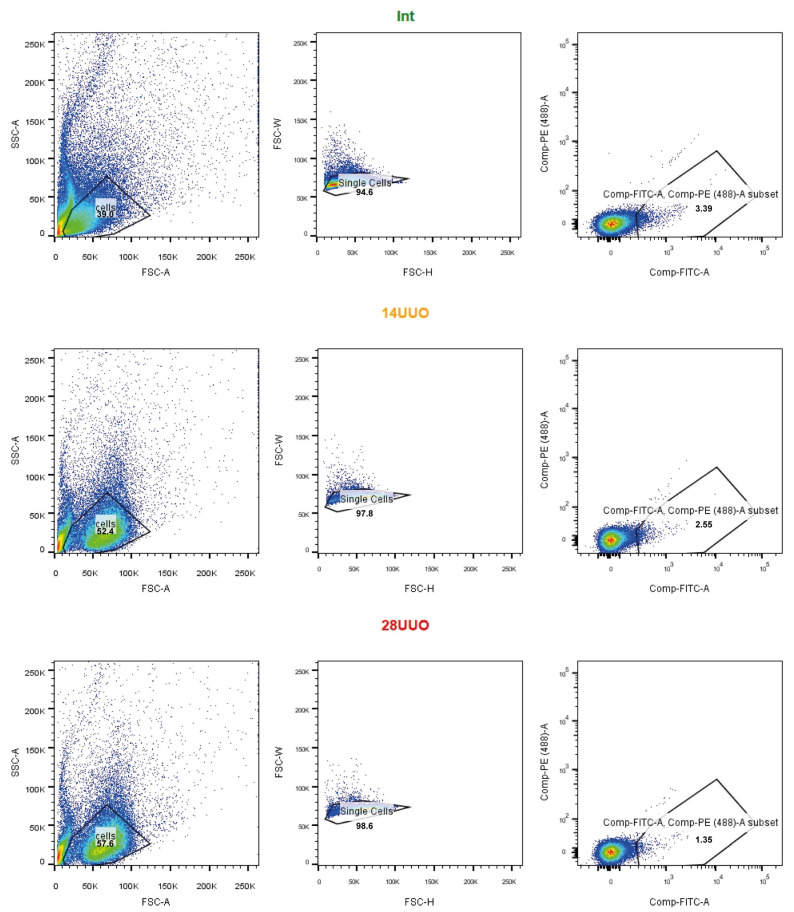
The GFP-positive cell population assessed in heart cell suspensions by FACS—the representative dot plots from the three experimental groups. Cells were consequently gated by light scatter—FSC-A vs. SSC-A; FSC-H vs. FSC-W—and fluorescence in the GFP channel. Cells were gated on GFP according to the non-GFP control.

**Figure A4:**
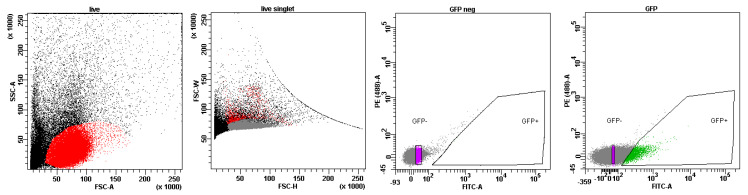
The gating strategy for cell sorting. Cells were consequently gated by light scatter based—FSC-A vs. SSC-A and FSC-H vs. FSC-W. The gate on the GFP-positive cells was set according to the non-GFP control. The gate on the GFP-negative population was set to distinguish cells with a 10-fold difference in median fluorescence intensity in the GFP channel. Cells from the GFP^+^ and GFP^−^ gates were sorted in parallel for further experiments.

Single-cell suspensions were prepared from the hearts of all experimental groups, using the MediMachine (BD, Franklin Lakes, NJ, USA), and passed through a 40 μm nylon mesh cell filter. For cell surface staining, the monoclonal antibodies anti-CD11b-PE-Cy5 (Invitrogen ThermoFisher Scientific, Waltham, MA, USA) were used. For this, the cells were incubated with the antibodies for 15 min at RT, washed with phosphate-buffered saline (PBS), and analyzed using a FACSAria SORP cell sorter (BD Biosciences, Franklin Lakes, NJ, USA). FlowJo v.X.0.7 software (FlowJo LLC, Ashland, OR, USA) was used for the data analysis. GFP^+^ and GFP^−^ cells were sorted using a FACSAria SORP cell sorter (BD Biosciences, Franklin Lakes, NJ, USA) with an 85 μm nozzle and corresponding pressure parameters. The gating strategy used to assess the population of GFP-positive cells and for sorting is shown in Figure A3 and Figure A4, respectively.

### 4.11. Statistical Analysis

Statistical analyses were performed with GraphPad Prism 10 (GraphPad Software Inc., La Jolla, CA, USA). The data were analyzed by parametric one-way ANOVA with Dunnett’s multiple comparison test based on their distribution normality (Shapiro–Wilk normality test). To identify and exclude outliers, we used Grubbs’ and ROUT tests. Data are presented as mean ± SEM.

## 5. Conclusions

In this study, we comprehensively characterized the morphofunctional alterations in the myocardium following UUO-induced RCS4 and elucidated the role of nestin-positive cells in this process using GFP–nestin transgenic mice. For the first time, we demonstrated that myocardial hypertrophy induced by a UUO of several weeks’ duration is accompanied by electrophysiological remodeling and increased titin content. Our findings suggest that the absence of myocardial fibrosis and inflammation may be attributed to the activation and expansion of nestin-positive cell populations. Notably, we provided the first phenotypic characterization of nestin-positive myocardial cells in UUO-induced cardiac dysfunction. Further investigation of the CPC subpopulation (nestin^+^TBX5^+^SCA-1^+^) may enhance therapeutic strategies for cardiovascular diseases.

## Figures and Tables

**Figure A2:**
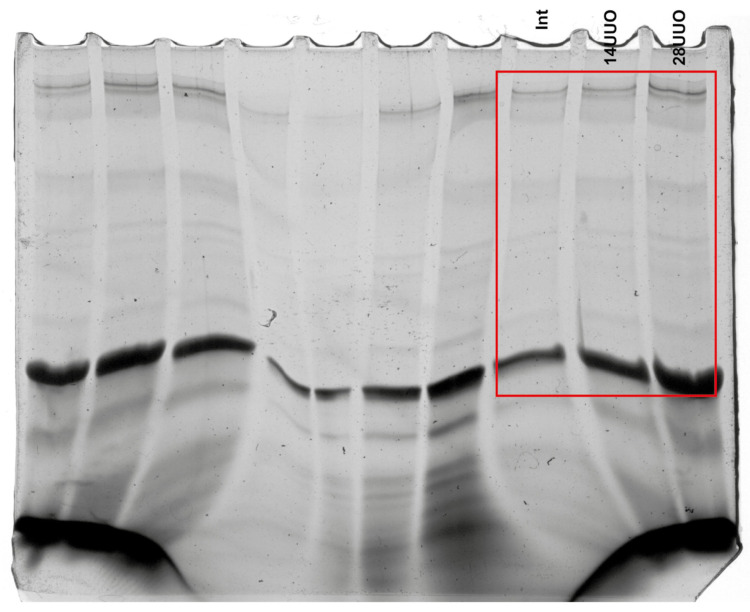
Raw images of titin electrophoresis gel shown in Figure 1D.

## Data Availability

The data that support the findings of this study are available from the corresponding author upon reasonable request.

## Abbreviations

The following abbreviations are used in this manuscript:

β1-AR	β1-adrenergic receptor
BP	Blood pressure
CK	Creatine kinase
CKD	Chronic kidney disease
c-Kit	KIT proto-oncogene receptor tyrosine kinase
COX2	Cyclooxygenase-2
CPCs	Cardiac progenitor cells
cx40	Connexin 40
CXCL	C-X-C motif chemokine ligand
ECG	Electrocardiogram
ECM	Extracellular matrix
FACS	Fluorescence-activated sorting
GFP	Green fluorescent protein
HR	Heart rate
IL1β	Interleukin 1β
LDH	Lactate dehydrogenase
L-NAME	N(G)-nitro-L-arginine methyl ester
MAP	Mean arterial pressure
MHC	Myosin heavy chains
MMP	Matrix metalloproteinase
PAS	Periodic acid–Schiff reagent
RCS 4	Renocardiac syndrome type 4
RT-PCR	Real-time PCR
SCA-1	Stem cell antigen-1
TBX5	T-box transcription factor 5
TGFβ1	Transforming growth factor β1
TIMP	Tissue inhibitor of matrix metalloproteinase
TNFα	Tumor necrosis factor α
vWF	von Willebrand factor
UUO	Unilateral ureteral obstruction

## Appendix A

**Table A1:** The number of mice in each experimental group.

Assays	Experimental Groups		
	Int	14UUO	28UUO
Morphometric analysis (heart)	11	8	8
Morphometric analysis (kidney)	4	4	5
ECG and blood pressure recording	8	8	7
Histochemical analysis	5	5	5
Titin electrophoresis	5	6	6
Plasma LDH and CK activity	13	12	12
RT-PCR (cardiac tissue, fibrosis markers)	7	9	10
RT-PCR (cardiac tissue, inflammation markers)	5	7	10
RT-PCR (cardiac tissue, *eGFP*)	7	7	8
RT-PCR (sorted cells)	5	3	3
Confocal microscopy	5	4	4
Flow cytometry	3	3	3

Notes: The value of “*n*” may be less than the number of mice indicated in the table due to the presence of outliers estimated by the ROUT and Grubbs test.

**Table A2:** Descriptive statistics for all bar graphs. Statistically significant changes are highlighted in red and bold.

Figure	Group	*p*-Value		Mean ± SD
		Int vs. 14UUO	Int vs. 28UUO	
Figure 1A	Int (*n* = 4)	0.99	**0.032**	0.71 ± 0.1
	14UUO (*n* = 4)			0.71 ± 0.12
	28UUO (*n* = 5)			0.53 ± 0.08
Figure 1B	Int (*n* = 11)	**0.044**	0.21	0.55 ± 0.03
	14UUO (*n* = 8)			0.61 ± 0.05
	28UUO (*n* = 8)			0.55 ± 0.06
Figure 1C	Int (*n* = 5) 1 dot represents 1 field of view (total: 428 fields)	**<0.0001**	**<0.0001**	15.9 ± 2.54
	14UUO (*n* = 5) 1 dot represents 1 field of view (total: 486 fields)			18.73 ± 2.71
	28UUO (*n* = 5) 1 dot represents 1 field of view (total: 480 fields)			17.42 ± 2.65
Figure 1D (T1/MHC)	Int (*n* = 5)	0.15	**0.02**	1.06 ± 0.19
	14UUO (*n* = 6)			1.24 ± 0.18
	28UUO (*n* = 6)			1.36 ± 0.08
Figure 1D (T2/MHC)	Int (*n* = 5)	0.45	0.1	0.57 ± 0.04
	14UUO (*n* = 6)			0.63 ± 0.12
	28UUO (*n* = 6)			0.69 ± 0.07
Figure 1D (T2/T1)	Int (*n* = 5)	0.91	0.98	0.5 ± 0.01
	14UUO (*n* = 6)			0.51 ± 0.03
	28UUO (*n* = 6)			0.51 ± 0.04
Figure 1D (TT/MHC)	Int (*n* = 5)	0.14	**0.024**	1.98 ± 0.34
	14UUO (*n* = 6)			2.32 ± 0.34
	28UUO (*n* = 6)			2.53 ± 0.21
Figure 1E (LDH)	Int (*n* = 13)	0.96	**0.039**	104.5 ± 25.14
	14UUO (*n* = 12)			100.6 ± 15.38
	28UUO (*n* = 12)			146.7 ± 50.19
Figure 1E (KK)	Int (*n* = 13)	0.59	0.4	105 ± 41.85
	14UUO (*n* = 12)			87.29 ± 16.66
	28UUO (*n* = 12)			128.7 ± 45.54
Figure 2A	Int (*n* = 8)	0.37	0.22	69.9 ± 5.6
	14UUO (*n* = 8)			73.6 ± 6.0
	28UUO (*n* = 7)			65.0 ± 6.1
Figure 2B	Int (*n* = 8)	0.57	0.16	28.4 ± 7.7
	14UUO (*n* = 8)			24.2 ± 9.6
	28UUO (*n* = 7)			20.18 ± 6.9
Figure 2C	Int (*n* = 8)	0.82	0.199	416 ± 54
	14UUO (*n* = 8)			394 ± 102
	28UUO (*n* = 7)			348 ± 66
Figure 2D	Int (*n* = 8)	0.54	0.11	145.1 ± 7.9
	14UUO (*n* = 8)			160.7 ± 37.1
	28UUO (*n* = 7)			178.6 ± 33.7
Figure 2F	Int (*n* = 8)	0.39	**0.012**	9.89 ± 0.83
	14UUO (*n* = 8)			10.62 ± 0.89
	28UUO (*n* = 7)			11.80 ± 1.80
Figure 2G	Int (*n* = 8)	0.76	0.69	22.78 ± 6.74
	14UUO (*n* = 8)			21.35 ± 2.54
	28UUO (*n* = 7)			24.53 ± 3.00
Figure 3A	Int (*n* = 7)	0.8	0.26	234.5 ± 138.5
	14UUO (*n* = 9)			261.2 ± 94.47
	28UUO (*n* = 10)			164.7 ± 56.96
Figure 3B	Int (*n* = 7)	0.13	0.89	4.08 ± 2.42
	14UUO (*n* = 9)			6.09 ± 2.43
	28UUO (*n* = 10)			4.52 ± 0.77
Figure 3C	Int (*n* = 7)	0.999	0.91	4.82 ± 2.85
	14UUO (*n* = 9)			4.79 ± 1.65
	28UUO (*n* = 10)			4.49 ± 1.26
Figure 3D	Int (*n* = 6)	0.18	**0.038**	49.29 ± 24.29
	14UUO (*n* = 9)			37.23 ± 8.58
	28UUO (*n* = 10)			31.87 ± 7.87
Figure 3E	Int (*n* = 6)	0.43	**0.017**	1 ± 0.66
	14UUO (*n* = 9)			0.76 ± 0.34
	28UUO (*n* = 10)			0.4 ± 0.22
Figure 3F	Int (*n* = 7)	**0.012**	0.71	0.67 ± 0.3
	14UUO (*n* = 9)			1.34 ± 0.56
	28UUO (*n* = 10)			0.82 ± 0.33
Figure 3G	Int (*n* = 7)	0.74	**0.083**	35.11 ± 13.63
	14UUO (*n* = 9)			31.94 ± 4.68
	28UUO (*n* = 10)			25.24 ± 9.03
Figure 4A	Int (*n* = 5)	**0.036**	**0.005**	12.21 ± 3.23
	14UUO (*n* = 7)			7.22 ± 3.56
	28UUO (*n* = 10)			5.63 ± 2.74
Figure 4B	Int (*n* = 5)	**0.008**	0.12	15.76 ± 5.5
	14UUO (*n* = 7)			5.54 ± 0.94
	28UUO (*n* = 10)			10.51 ± 5.19
Figure 4C	Int (*n* = 5)	0.44	0.21	0.2 ± 0.04
	14UUO (*n* = 7)			0.25 ± 0.09
	28UUO (*n* = 10)			0.13 ± 0.05
Figure 4D	Int (*n* = 5)	0.99	0.57	0.34 ± 0.14
	14UUO (*n* = 7)			0.34 ± 0.12
	28UUO (*n* = 10)			0.42 ± 0.2
Figure 4E	Int (*n* = 5)	0.59	0.93	0.34 ± 0.09
	14UUO (*n* = 7)			0.27 ± 0.14
	28UUO (*n* = 10)			0.31 ± 0.12
Figure 4F	Int (*n* = 5)	0.92	0.46	1.58 ± 0.91
	14UUO (*n* = 7)			1.76 ± 1.04
	28UUO (*n* = 10)			1.06 ± 0.54
Figure 4G	Int (*n* = 3)	**0.002**	**0.002**	2.18 ± 0.73
	14UUO (*n* = 3)			0.22 ± 0.06
	28UUO (*n* = 3)			0.13 ± 0.04
Figure 5A	Int (*n* = 7)	0.16	**0.0482**	370 ± 65.7
	14UUO (*n* = 7)			639 ± 183
	28UUO (*n* = 8)			716 ± 341
Figure 10A	Int (*n* = 3)	0.09	0.58	12.37 ± 6.8
	14UUO (*n* = 3)			23.1 ± 2.96
	28UUO (*n* = 3)			9.249 ± 8.37
Figure 10B	Int (*n* = 5)	0.95	0.37	9.94 ± 6.28
	14UUO (*n* = 3)			8.8 ± 6.34
	28UUO (*n* = 3)			4.28 ± 4.29
Figure 10C	Int (*n* = 3)	0.84	0.77	3.57 ± 2.78
	14UUO (*n* = 3)			2.46 ± 0.27
	28UUO (*n* = 3)			2.19 ± 3.8

Notes: The value of “*n*” may be less than the number of mice indicated in the table due to the presence of outliers estimated by the ROUT and Grubbs test.

**Table A3:** Sequences of primers used for gene expression estimation.

Gene Name	Primer Nucleotide Sequence (5′ to 3′)	PCR Product Size, bp	Genbank Accession Number
*eGFP*	for CACGACTTCTTCAAGTCCGC	318	U55762.1
	rev GGTGTTCTGCTGGTAGTGGT		
*Col1a1*	for CGATGGATTCCCGTTCGAGT	197	NM_007742.4
	rev CGATCTCGTTGGATCCCTGG		
*Col4a1*	for ATGGCTTGCCTGGAGAGATAGG	134	NM_009931.2
	rev TGGTTGCCCTTTGAGTCCTGGA		
*Tgfb1*	for TGATACGCCTGAGTGGCTGTCT	107	NM_011577.2
	rev CACAAGAGCAGTGAGCGCTGAA		
*Timp1*	for GCAACTCGGACCTGGTCATAA	226	NM_011593.2
	rev CGGCCCGTGATGAGAAACT		
*Timp2*	for TCAGAGCCAAAGCAGTGAGC	142	NM_011594.3
	rev GCCGTGTAGATAAACTCGATGTC		
*Mmp2*	for CAAGTTCCCCGGCGATGTC	171	NM_008610.3
	rev TTCTGGTCAAGGTCACCTGTC		
*Mmp9*	for CTGGACAGCCAGACACTAAAG	145	NM_013599.5
	rev CTCGCGGCAAGTCTTCAGAG		
*Cxcl1*	for CACCTCAAGAACATCCAGAGCT	163	NM_008176.3
	rev ACTTGGGGACACCTTTTAGCAT		
*Cxcl9*	for ATCATCTTCCTGGAGCAGTGTG	193	NM_008599.4
	rev CTAGGCAGGTTTGATCTCCGTT		
*Il1b*	for TTGAAGAAGAGCCCATCCTCTG	144	NM_008361.4
	rev CTTTCAGCTCATATGGGTCCGA		
*Il6*	for ACATAAAATAGTCCTTCCTACCCCA	100	NM_031168.2
	rev GATGAATTGGATGGTCTTGGTCC		
*Tnfa*	for CCAAAGGGATGAGAAGTTCCCA	249	NM_013693.3
	rev ACCTGGGAGTAGACAAGGTACA		
*Cox2*	for CTGACCCCCAAGGCTCAAATAT	224	NM_011198.5
	rev GGGATACACCTCTCCACCAATG		
*C-kit*	for TTTGCTGAGCTTCTCCTACCAG	82	NM_001122733.1
	rev TCCCATAGGACCAGACATCACT		
*Sca-1*	for GAGACTTCTTGCCCATCAATTACC	82	NM_001271416.1
	rev GAGAATCCACAATAACTGCTGCC		
*Tbx5*	for CCAAAGACAGGTCTTGCGATTCG	192	NM_011537.3
	rev TTCTCCTCCCTGCCTTGGTGAT		
*Rplp0*	for GCTTCGTGTTCACCAAGGAGGA	135	NM_007475.5
	rev GTCCTAGACCAGTGTTCTGAGC		

Supplementary Methods

- Measurement of serum urea concentration

The serum urea concentration was measured spectrophotometrically using the diacetylmonoxime method with commercial reagent kits (AgatMed, Moscow, Russia) according to the manufacturer’s protocol. Absorbance changes at 540 nm were measured using a PE-5400UV spectrophotometer (Ekroskhim, Moscow, Russia).

- Determination of Oxidative Stress Marker Concentrations

Kidney tissue samples were homogenized in ice-cold PBS using a glass homogenizer at a 1:10 (*w*/*v*). The homogenates were centrifuged at 3000× *g* for 10 min at 4 °C to obtain supernatants for analysis. Lipid peroxidation was assessed by measuring thiobarbituric acid reactive substances (TBARS) according to the method described by [65]. Briefly, samples were reacted with thiobarbituric acid, and absorbance was measured at 532 nm using a PE-5400UV spectrophotometer (Ekroskhim, Russia). Reduced glutathione (GSH) levels were determined using Ellman’s reagent [66]. The reaction product’s absorbance was read at 412 nm on the same PE-5400UV spectrophotometer.
